# Insights Into Gut Barrier Dysfunction and Metabolic Alterations in Mycophenolate‐Induced Enteropathy

**DOI:** 10.1111/bcpt.70204

**Published:** 2026-02-01

**Authors:** Clarisse Brossier, François‐Ludovic Sauvage, Christel Valencia‐Schmitt, Valérie Calco, Pascal Villa, Pierre Marquet, Roland Lawson

**Affiliations:** ^1^ INSERM, Univ. Limoges Pharmacology & Transplantation, U1248 Limoges France; ^2^ Strasbourg Integrative Biological Chemistry Platform (PCBIS), UAR 3286 CNRS–University of Strasbourg Strasbourg Institute for drug discovery and development (IMS) Illkirch France

**Keywords:** Caco‐2 cells, gut epithelial barrier, lucifer yellow, MPA‐induced enteropathy, mycophenolic acid (MPA), targeted metabolomics, transepithelial electrical resistance

## Abstract

Mycophenolic acid (MPA) is a widely used immunosuppressant whose use is often limited by gastrointestinal toxicity. Gut bacterial hydrolysis of liver‐derived MPA glucuronides increases local exposure to MPA, potentially impairing epithelial barrier function and cellular metabolism. To explore the effects of MPA on gut barrier integrity and metabolic pathways in gut epithelial cells, Caco‐2 cells were exposed to MPA (10 or 100 μM), and barrier function was assessed by transepithelial electrical resistance (TEER) and lucifer yellow (LY) permeability in both differentiated and early‐stage monolayers, while intracellular metabolic changes were investigated using targeted LC–MS/MS metabolomics. In differentiated monolayers, MPA did not significantly alter LY transport or TEER measurements. In contrast, MPA exposure during the early stages of monolayer formation reduced TEER values, 3 days after MPA withdrawal (Day 6; *p* < 0.01). The effects of 100‐μM MPA were still noticeable at Day 10, as confirmed by LY permeability (*p* < 0.05). Metabolomic profiling clearly separated exposed from control cells (PCA, PC1 + PC2 = 92% variance). At 10 and 100 μM, 9 and 8 metabolites were significantly altered, with 6 common to both doses. Pathway enrichment revealed perturbations mainly in nucleotide synthesis, consistent with altered metabolic activity.

## Plain Language Summary

Mycophenolic acid (MPA) is an important drug used to prevent organ rejection, but it is linked to gastrointestinal side effects. In this study, we used a digestive cell line to examine how MPA affects barrier function and cellular metabolism. Mature, differentiated cell layers were mostly unchanged, but early‐stage cell layers became weaker after MPA exposure, even days after the drug was removed. We also detected distinct metabolic changes, especially in pathways involved in nucleotide production. Overall, these findings suggest that MPA may disrupt vulnerable gut cells by weakening their barrier and altering key metabolic processes.

**Abbreviations**
DMEM: Dulbecco's modified Eagle's medium
DMSO: Dimethylsulfoxide
HBSS: Hank's balanced salt solution
IMPDH: Inosine‐5′‐monophosphate dehydrogenase
LC–MS/MS: liquid chromatography coupled to tandem mass spectrometry
LY: lucifer yellow
MPA: mycophenolic acid
MPAG: MPA‐7‐O‐glucuronide
MRM: multireaction monitoring
PCA: principal component analysis
SEM: standard error of the mean
TEER: transepithelial electrical resistance

## 1 Introduction

Gastrointestinal disorders associated with mycophenolic acid (MPA) limit the therapeutic benefits of this widely used immunosuppressant. In transplant patients, these disorders manifest as nausea, vomiting, diarrhoea and, in severe cases, bleeding ulcerations [1, 2]. Histological lesions often resemble those observed in acute colitis or inflammatory bowel disease, including crypt distortion, cell apoptosis and mucosal architectural disorganization, accompanied by lamina propria inflammation [2, 3]. Such MPA‐induced enteropathy can compromise patient adherence to therapy and adversely affect graft survival [4], but its underlying mechanisms remain elusive.

Recent evidence highlights the role of gut dysbiosis and local accumulation of MPA, suggesting a complex multifactorial pathogenesis [5, 6, 7]. MPA is a potent, selective, non‐competitive and reversible inhibitor of inosine‐5′‐monophosphate dehydrogenase (IMPDH), a key enzyme in de novo purine nucleotide synthesis and DNA biosynthesis in T‐ and B‐lymphocytes [8].

Clinically, MPA is administered as a prodrug (mycophenolate mofetil) or as mycophenolate sodium. In the liver, MPA is metabolized by UDP‐glucuronosyltransferases into its major inactive metabolite, MPA‐7‐O‐glucuronide (MPAG), which is excreted in urine but is partly secreted into bile [9, 10, 11]. Gut bacterial β‐glucuronidases can reactivate MPAG into MPA, contributing to local epithelial exposure, partly followed by enterohepatic circulation [12]. Pharmacological inhibition of bacterial β‐glucuronidases in mice reduced MPA reactivation and prevented architectural disorganization of the proximal colon mucosa, supporting the direct role of MPA in gut epithelial toxicity [7, 13, 14]. Beyond its immunosuppressive function, MPA exhibits broad antifungal, antibacterial and antiviral properties [8, 9]. Preclinical studies indicate that it can alter tight junction proteins (ZO‐1, occludins and claudins) and impair intestinal permeability [15, 16]. The intestinal lining regulates nutrient absorption while preventing the translocation of pathogens and microbial metabolites into the systemic circulation, a function largely maintained by enterocytes, which account for over 80% of the epithelial layer [17, 18]. The barrier's structure, composed of villi and crypts, relies on a differentiation gradient and renewal of epithelial cells every 4–5 days, which are essential for preserving barrier integrity [18, 19, 20, 21].

Dysfunction of this barrier facilitates the systemic dissemination of luminal compounds such as bacterial lipopolysaccharides, promoting chronic low‐grade inflammation, endothelial activation and cardiovascular complications [22].

In this study, we investigated the direct effects of MPA on the gut epithelial barrier integrity and cell physiology using permeability assays and targeted metabolomics, aiming to provide mechanistic insights into the pathways underlying MPA‐induced enteropathy.

## 2 Materials and Methods

### 2.1 Experimental Design

The study was conducted in accordance with the Basic & Clinical Pharmacology & Toxicology policy for experimental studies [23]. Caco‐2 cells (ATCC), a well‐characterized human colorectal adenocarcinoma‐derived model of gut epithelial cells with epithelial morphology, were used to study gut barrier homeostasis [19, 24].

**FIGURE 1:**
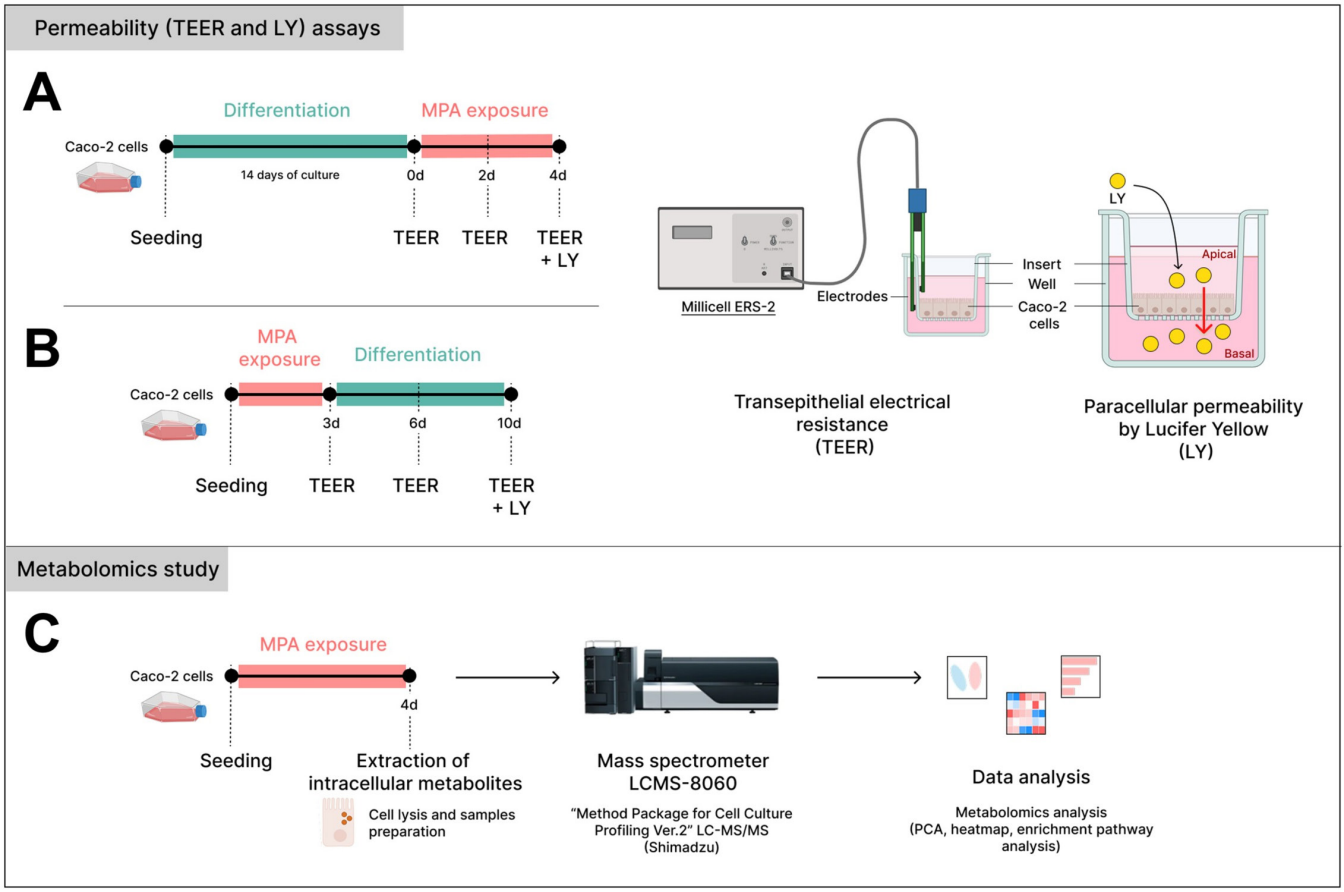
Experimental design for permeability assays and metabolomics analysis in Caco‐2 cells. (A) Caco‐2 cells were differentiated for 14 days and then exposed to MPA (10 or 100 μM) or vehicle (0.5% DMSO) for 4 days. (B) Caco‐2 cells were exposed to MPA (10 or 100 μM) or vehicle (0.5% DMSO) during the seeding phase and maintained for 3 days. The MPA‐containing medium was then replaced with standard culture medium, and cells were maintained for an additional 7 days. Transepithelial electrical resistance (TEER) was measured at multiple time points in both experimental procedures. Paracellular permeability was assessed at the end of each procedure by measuring lucifer yellow (LY) transport after a 2‐h incubation. LY permeability was expressed as the basolateral‐to‐apical fluorescence ratio. (C) For the metabolomics analysis, Caco‐2 cells were exposed to MPA (10 or 100 μM) or vehicle (0.5% DMSO) for 4 days. Intracellular metabolites were extracted and analysed by LC–MS/MS, and data processing was performed using MetaboAnalyst software.

We assessed the effects of MPA on (i) the integrity of the gut epithelial barrier in differentiated Caco‐2 monolayers (Figure 1A); (ii) the ability of Caco‐2 cells at the early stage to restore epithelial density after MPA exposure (Figure 1B); and (iii) intracellular metabolic changes following MPA exposure (Figure 1C). MPA concentrations of 10 and 100 μM were chosen based on previously reported in vitro studies assessing gut epithelial responses to MPA [15, 16] and on faecal levels reported in mouse models of MPA‐induced enteropathy [6, 7].

### 2.2 Cell Culture

Caco‐2 cells were cultured in Dulbecco's Modified Eagle's Medium (DMEM, 4.5 g/L glucose, pyruvate; Gibco), supplemented with 10% heat‐inactivated foetal bovine serum (FBS; Gibco) and 1% penicillin/streptomycin (Gibco). Cells were incubated at 37°C in 5% CO_2_ and subcultured twice per week using 0.25% trypsin solution (Gibco) in 75‐cm^2^ flasks.

For transepithelial electrical resistance (TEER) measurement and the lucifer yellow (LY) permeability assay, cells were seeded on Transwell inserts (pore size: 0.4 mm, culture surface 0.33 cm^2^). For metabolomics analyses, cells were seeded at confluence (300 000 cells/well) in 12‐well plates.

### 2.3 Permeability (TEER and LY) Assays

TEER was used to assess gut epithelial barrier integrity. Caco‐2 cells were exposed after differentiation (Figure 1A) or seeding (Figure 1B) to 10 or 100 μM of MPA (Sigma‐Aldrich) dissolved in dimethyl sulfoxide (DMSO) to a final concentration of 0.5%. Solutions were applied to both the apical and basolateral compartments of the inserts. TEER was measured using a Millicell ERS‐2 voltohmmeter with STX3 electrodes (Sigma‐Aldrich). Prior to the first measurement, culture media were replaced with MPA solutions on Day 0 (Figure 1A) or with standard culture medium on Day 3 (Figure 1B), and plates were equilibrated at room temperature for 1 h. TEER was further recorded on different days (Figure 1A,B), with three measurements performed per well at each time point for each condition. A cell‐free insert was used as a background control (resistance = 100 Ω), and this value was subtracted from each reading. Three independent experiments (*n* = 3) were performed with three replicates for each condition. The mean of three readings per well was used for statistical analysis.

At the end of the procedure (on Days 4 or 10, Figure 1A,B), passive paracellular transport was assessed using LY (Sigma‐Aldrich). Culture media was replaced with Hanks' balanced salt solution (HBSS, Gibco), and LY was added to the apical compartment at a final concentration of 10 μM. Plates were incubated at 37°C in 5% CO_2_. After 2 h, samples were then collected from both the apical and basolateral compartments and analysed by fluorescence (excitation: 405 nm; emission: 535 nm). Three independent experiments (*n* = 3) were performed with three replicates for each condition. The amount of LY that crossed the epithelial monolayer was quantified by fluorescence, based on the reading of each well, and results were expressed as the basolateral‐to‐apical fluorescence ratio.

### 2.4 Metabolomics Study

#### 2.4.1 Intracellular Metabolite Extraction

Caco‐2 cells were exposed to 10 or 100 μM of MPA for 4 days and compared to the control condition (0.5% DMSO). Intracellular metabolites were extracted following a previously published protocol [25], with slight modifications. Cells were washed with cold PBS (4°C) and lysed for 20 min at −80°C in 2 mL of a methanol/water solution (80:20, v/v) containing the internal standard (2‐isopropylmalic acid at 0.5 mmol/L, diluted 1:1000), previously stored at −80°C. Cells were scraped, and lysates centrifuged at 20 000 g for 6 min at 4°C. Supernatants (1.5 mL) were collected and dried under vacuum. Lyophilized extracts were reconstituted in 110 μL of ultrapure water and transferred to vials for liquid chromatography coupled to tandem mass spectrometry (LC–MS/MS) analysis.

#### 2.4.2 LC–MS/MS Analysis

Samples (3 μL each) were analysed using a Shimadzu LC–MS/MS‐8060 triple quadrupole system with the ‘Cell Culture Profiling Ver. 2’ package (Shimadzu). Targeted metabolites included amino acids, bi‐ and tripeptides, nucleic acid‐related compounds, vitamins and carbohydrates.

Briefly, chromatographic separation was performed using a Discovery HS F5, 3‐μm particle size, 15 cm × 2.1 mm column (Sigma‐Aldrich) kept at 40°C. The mobile phase was made of solvents A (0.1% formic acid in water) and B (0.1% formic acid in acetonitrile). The flow rate was set to 350 μL/min with the following linear mobile phase gradient: 0–1.4 min: 0% B; 1.4–3.5 min: 0% to 25% B; 3.5–7.5 min: 25% to 35% B; 7.5–10.3 min: 35% to 95% B; 10.3–13.7 min: hold at 95% B; 13.7–13.8 min: return to 0% B; 13.8–17 min: stabilization at 0% B. Detection was performed in the scheduled multireaction monitoring (MRM) mode, alternating positive and negative electrospray ionization. Identification was based on relative retention times (normalized to the internal standard) and transition intensity ratios. Semi‐quantification was based on the ratio of the selected MRM peak area to that of the internal standard.

#### 2.4.3 Metabolomics Data Processing and Analysis

Metabolomic data processing was performed using MetaboAnalyst 6.0 from data of three independent experiments (*n* = 3). For each, three replicates were performed where the metabolites were quantified, and the area ratio was calculated. Principal component analysis (PCA), hierarchical clustering (heatmap) and volcano plots were generated. Significantly altered metabolites were identified by volcano plot using the following parameters: *p* < 0.05 and fold‐change threshold ≥ 2. Pathway enrichment analysis was conducted using the 80‐metabolite KEGG‐based human metabolic pathway library. Pathways were visualized using R Studio.

### 2.5 Statistical Analysis

TEER and LY data were analysed using GraphPad Prism 9 (San Diego, California, USA, www.graphpad.com) and were presented as mean ± standard error of the mean (SEM). Normality of the data was assessed using the Shapiro–Wilk test. One‐ or two‐way ANOVAs were applied when appropriate, followed by Bonferroni post hoc tests if the ANOVA indicated overall significance (*p* < 0.05).

## 3 Results

### 3.1 MPA Increases Gut Epithelial Cell Permeability at the Higher Concentration

**FIGURE 2:**
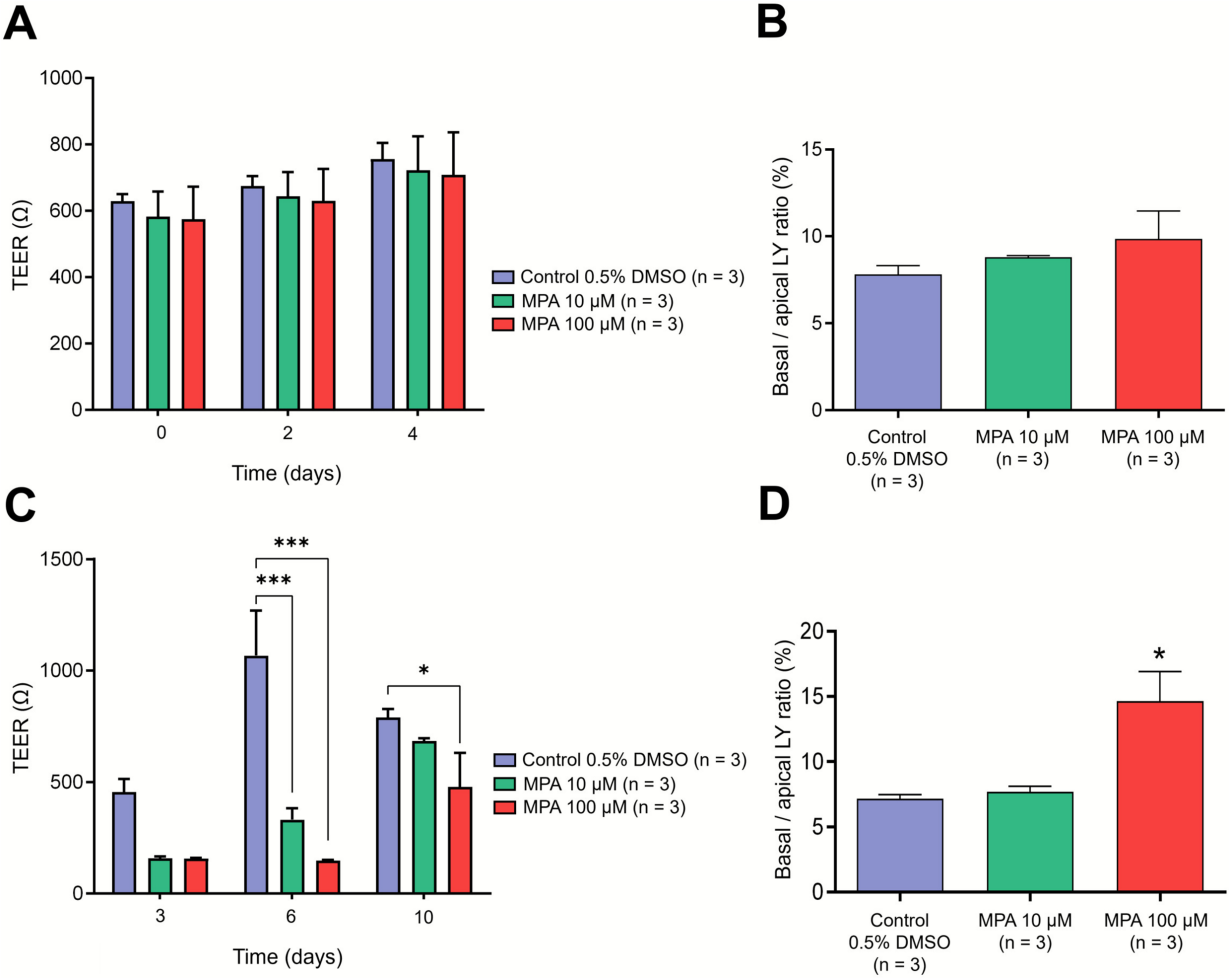
Mycophenolic acid reduces barrier integrity in Caco‐2 intestinal epithelial cells. (A) Caco‐2 cells were differentiated for 14 days and then exposed to MPA (10 or 100 μM) or vehicle (0.5% DMSO) for 4 days. Transepithelial electrical resistance (TEER) was measured at 0, 2 and 4 days. (B) Paracellular permeability was assessed after 96 h by measuring lucifer yellow (LY) transport following a 2‐h incubation, expressed as the basolateral‐to‐apical fluorescence ratio. (C) Caco‐2 cells were exposed to MPA (10 or 100 μM) or vehicle (0.5% DMSO) during the seeding phase and maintained for 3 days. The MPA‐containing medium was then replaced with standard culture medium, and cells were maintained for an additional 7 days. TEER was measured on Days 3 (end of MPA exposure), 6 and 10 (after 3‐ and 7‐day post‐MPA removal, respectively). (D) Paracellular permeability was assessed on Day 10 by measuring LY transport after a 2‐h incubation. Data are presented as mean ± SEM from three independent experiments (*n* = 3), with three replicates per condition. For the TEER experiment, the mean of the three readings per well was used for statistical analysis. Two‐way ANOVA for TEER measurements was performed (A, C), and one‐way ANOVA for LY permeability assays (B, D) followed by Bonferroni's post hoc test when overall significance was detected (*p* < 0.05). *p < 0.05; ***p < 0.001 versus control (Bonferroni post hoc test).

Incubation of differentiated Caco‐2 cells with 10‐ and 100‐μM MPA for 4 days did not significantly affect epithelial barrier density, as assessed by TEER measurement (control: 756 ± 49 Ω; MPA [10 μM]: 722 ± 103 Ω; MPA [100 μM]: 708 ± 128 Ω; two‐way ANOVA, *p* > 0.05; Figure 2A). Similarly, the higher MPA concentration (100 μM) induced a slight, nonsignificant increase in LY paracellular transport compared to control (control: 7.82 ± 0.50; MPA [10 μM]: 8.81 ± 0.09; MPA [100 μM]: 9.86 ± 1.61; one‐way ANOVA, *p* > 0.05; Figure 2B).

In contrast, exposure of Caco‐2 cells to MPA (10 or 100 μM) during the cell adhesion stage resulted even if it is nonsignificant, in a marked threefold reduction in TEER values at Day 3 (control: 456 ± 58 Ω; MPA [10 μM]: 159 ± 7 Ω; MPA [100 μM]: 157 ± 3 Ω; two‐way ANOVA, *p* > 0.05; Figure 2C). This effect became significant at Day 6 (i.e., 3 days after MPA withdrawal; control: 1067 ± 202 Ω; MPA [10 μM]: 331 ± 52 Ω; MPA [100 μM]: 147 ± 5 Ω; two‐way ANOVA, *p* < 0.01; Bonferroni post hoc test, control versus MPA [10] or MPA [100], *p* < 0.001; Figure 2C). By Day 10, TEER values in cells exposed to 10‐μM MPA were no longer significantly different from control, whereas cells exposed to 100‐μM MPA still exhibited reduced barrier integrity (control: 790 ± 39 Ω; MPA [10 μM]: 684 ± 13 Ω; MPA [100 μM]: 479 ± 152 Ω; two‐way ANOVA, *p* < 0.01; Bonferroni post hoc test, control versus MPA [100], *p* < 0.05; Figure 2C). These findings were further supported by LY paracellular permeability analysis at the end of the experiment. LY transport was approximately doubled in the 100‐μM MPA condition compared to control, whereas LY permeability in cells treated with 10‐μM MPA did not differ significantly from control (control: 7.17 ± 0.31; MPA [10 μM]: 7.71 ± 0.41; MPA [100 μM]: 14.60 ± 2.27; one‐way ANOVA, *p* < 0.05; Bonferroni post hoc test, control versus MPA [100], *p* < 0.05; Figure 2D), consistent with TEER measurements at this concentration.

### 3.2 MPA Reshapes the Intracellular Metabolomic Profile of Gut Epithelial Cells

**FIGURE 3:**
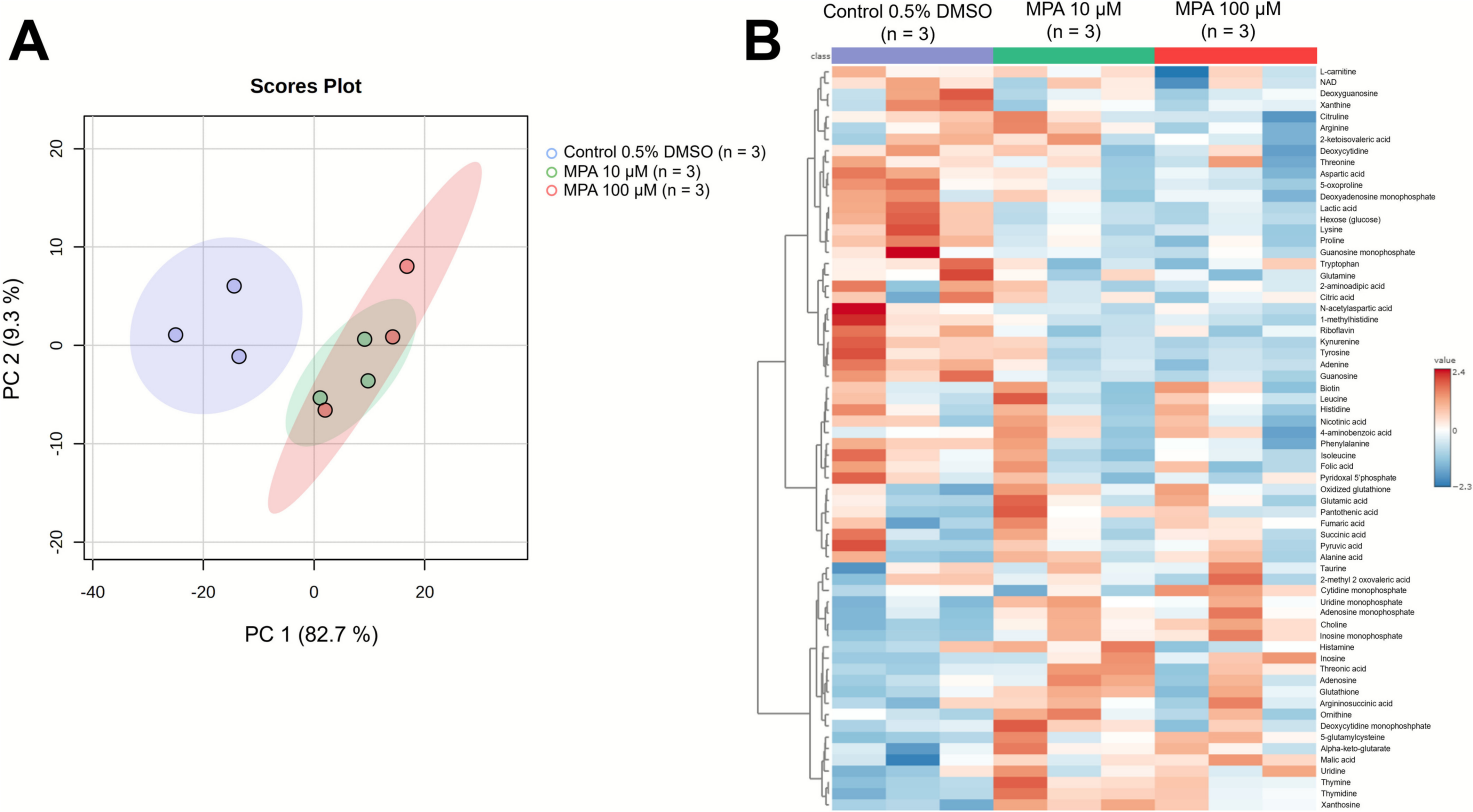
Multivariate analysis of intracellular metabolites in Caco‐2 cells detected by LC–MS/MS. (A) Principal component analysis (PCA) separates the three experimental conditions: control (blue), MPA 10 μM (green) and MPA 100 μM (red), based on the principal components PC1 (82.7%) and PC2 (9.3%). (B) Hierarchical clustering heatmap of intracellular metabolite profiles across the three experimental conditions. A total of 66 intracellular metabolites were detected. Data from three independent experiments (*n* = 3) were used. For each, three replicates were performed. Metabolites were analytically detected and quantified, and the surface area ratio was calculated.

PCA clearly separated Caco‐2 cells exposed to MPA (10 and 100 μM) from control cells (0.5% DMSO) based on principal components PC1 and PC2, which accounted for 82.7% and 9.3% of the total variance, respectively (Figure 3A). A hierarchical clustering heatmap based on 66 intracellular metabolites detected by LC–MS/MS revealed distinct metabolomic profiles following exposure to both concentrations of MPA compared to the control condition (Figure 3B).

### 3.3 Distinct and Overlapping Metabolomic Signatures at 10‐ and 100‐μM MPA

**FIGURE 4:**
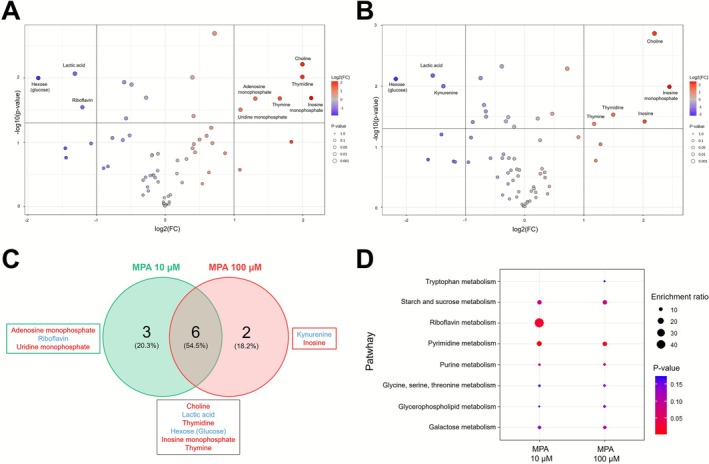
Intracellular metabolomic profile of Caco‐2 cells exposed to 10‐ and 100‐μM MPA. Volcano plots illustrate significantly altered metabolites in Caco‐2 cells after incubation with (A) 10 μM or (B) 100 μM of MPA. Blue dots indicate decreased metabolite levels; red dots indicate increased levels. Analyses used an unpaired *t*‐test with fold‐change threshold (FC) = 2 and *p* < 0.05, comparing MPA‐exposed cells to control (0.5% DMSO). Of the 66 intracellular metabolites identified, exposure to 10‐ and 100‐μM MPA significantly altered the levels of 9 and 8 metabolites, respectively. (C) Venn diagram showing overlap and unique metabolites affected at each concentration: 6 common, 3 exclusive to 10 μM and 2 exclusives to 100 μM. Metabolites shown in blue indicate a decrease, while those in red indicate an increase. (D) Dot plot showing pathway enrichment analysis of intracellular metabolites significantly altered in Caco‐2 cells exposed to MPA at 10 and 100 μM versus control (0.5% DMSO). Enrichment analysis was performed using a curated set of 80 human metabolic pathways from the Kyoto Encyclopedia of Genes and Genomes (KEGG).

Volcano plot analysis identified significant alterations in metabolite abundance, with 9 and 8 metabolites significantly affected at 10 and 100 μM (*p* < 0.05 and FC = 2; Figure 4A,B). The Venn diagram evidenced that six metabolites were commonly altered at both MPA concentrations. Among the metabolites modulated by 10‐μM MPA, six were significantly upregulated (choline, thymidine, inosine monophosphate, thymine, adenosine monophosphate and uridine monophosphate) and three downregulated (lactic acid, hexose [glucose] and riboflavin) (*p* < 0.05 and FC = 2; Figure 4C). At 100 μM, five metabolites were significantly upregulated (choline, inosine monophosphate, thymidine, inosine and thymine) and three downregulated (hexose [glucose], lactic acid and kynurenine) under the same statistical thresholds (Figure 4C).

### 3.4 MPA‐Induced Metabolic Rewiring of Caco‐2 Cells Targets Core Cellular Pathways

Pathway enrichment analysis revealed that MPA exposure significantly affected key metabolic pathways in Caco‐2 cells, including those involved in purine and pyrimidine metabolism, as well as riboflavin metabolism. Significantly altered metabolites, such as choline and kynurenine, are involved in amino acid metabolism (i.e., glycine, serine, threonine and tryptophan metabolism). Alterations in galactose and starch/sucrose metabolism were observed through modifications in lactic acid and glucose metabolites (Figure 4D).

**FIGURE 5:**
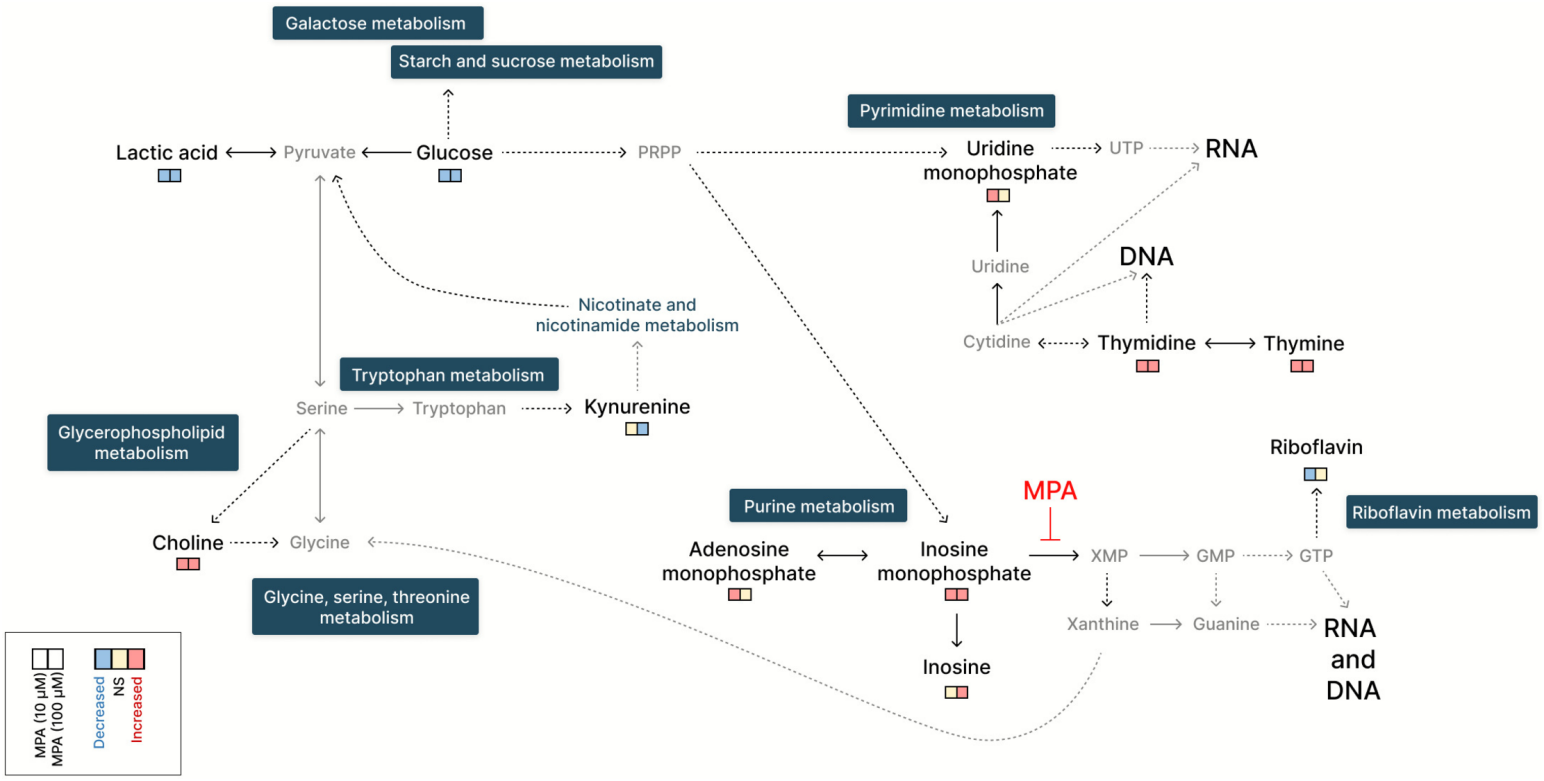
Metabolic nodes and networks affected by MPA in Caco‐2 cells. Metabolic networks map indicating detected metabolites (black) and undetected but relevant intermediates (grey) within the key pathways affected by MPA. Dotted arrows represent multiple intermediate metabolites between nodes. Colour‐coded squares represent changes in metabolite levels for each MPA concentration relative to control: decreased (light blue), increased (pink) and no significant change (beige). GMP, guanosine monophosphate; GTP, guanosine triphosphate; PRPP, phosphoribosylpyrophosphate; UTP, uridine triphosphate; XMP, xanthosine monophosphate.

The edges between pathways indicate functional and biochemical crosstalk, suggesting that MPA does not act on isolated nodes but rather perturbs the interconnected metabolic network (Figure 5).

## 4 Discussion

In this study, we comprehensively evaluated the effects of MPA on a gut epithelial cell line using complementary barrier integrity assays and targeted metabolomics. Our findings reveal, for the first time, that MPA preferentially affects cells during the early stages of gut epithelial monolayer formation while simultaneously altering metabolic activity. This dual impact provides a mechanistic explanation for MPA‐induced enteropathy in transplant patients.

### 4.1 MPA Disrupts the Gut Epithelial Barrier Function

We demonstrate that MPA, even at high concentration (100 μM), did not significantly increase paracellular permeability in differentiated Caco‐2 cells, contrary to previous studies reporting reduced TEER and increased FITC‐dextran flux [15]. Critically, Caco‐2 cells at the early stage of monolayer formation were more susceptible to MPA effects: exposure to 10 and 100 μM during adhesion reduced TEER by around 65% at Day 3, and at the higher concentration, continued to impair barrier integrity even after 10 days, indicating a persistent defect in epithelial regeneration.

These results suggest that MPA preferentially targets proliferating enterocytes, compromising their density and renewal potential, which is essential for maintaining intestinal homeostasis [18]. Mechanistically, such barrier dysfunction is likely mediated by downregulation of tight junction proteins, including ZO‐1, occludin and claudins, thereby increasing paracellular flux [15, 16, 18, 26, 27, 28].

### 4.2 MPA Induces Metabolic Alterations That Exacerbate Barrier Vulnerability

Targeted metabolomics revealed significant alterations in purine and pyrimidine metabolism. Notably, inosine monophosphate and thymidine were upregulated, suggesting compensatory activation of pyrimidine synthesis in response to impaired guanosine production. These findings are consistent with the known mechanism of MPA, which inhibits IMPDH [8]. This inhibition blocks the conversion of inosine monophosphate to xanthine‐5‐phosphate, and consequently to guanosine‐5‐phosphate, preferentially by targeting the inducible IMPDH2 isoform [29]. This selectivity underlies its immunosuppressive efficacy through inhibition of lymphocyte proliferation. However, gut epithelial cells predominantly express the constitutive IMPDH1 isoform [30, 31], historically thought to confer lower sensitivity to MPA. Our results, showing marked metabolic disruption in these cells, challenge this view and suggest that IMPDH1 may also contribute to MPA's pharmacological and clinical effects. Supporting this notion, a pharmacogenetic study reported that an IMPDH1 polymorphism was associated with a reduced risk of acute rejection 1‐year post‐transplantation, highlighting the isoform's potential clinical relevance [32].

Concomitantly, depletion of glucose and lactic acid in response to MPA exposure indicates high energy consumption in Caco‐2 cells and may limit nucleic acid synthesis, further reducing enterocyte proliferation and renewal capacity. Kynurenine, a key metabolite in tryptophan metabolism, was significantly decreased following exposure to 100 μM of MPA. Tryptophan metabolism plays a crucial role in intestinal homeostasis [33], and two kynurenine‐derived metabolites (e.g., kynurenic acid and xanthurenic acid) were found to be negatively correlated with the severity of inflammation in a mouse model of DSS‐induced colitis. These metabolites were also found to promote the proliferation of human gut epithelial cells [34]. These observations support the decrease of kynurenine as a potential contributor to increased intestinal epithelial cell permeability.

Choline, a precursor of phosphatidylcholine, essential for proliferating cells and a major component of cellular membranes [35], was significantly increased in Caco‐2 cells after MPA exposure. Choline has been demonstrated to enhance cell proliferation and reduce apoptosis through Bcl‐2 upregulation in porcine intestinal cells [36] and to attenuate oxidative stress in bovine mammary epithelial cells [37]. In vivo, dietary choline deficiency was associated with intestinal inflammation, characterized by upregulated pro‐inflammatory cytokines and decreased mRNA levels of tight junction proteins in juvenile Jian carp. However, excessive dietary choline intake (1820 mg/kg) may have controversial effects on the intestinal barrier [38].

Riboflavin decreased following MPA exposure at 10 μM but not at 100 μM. Riboflavin depletion in intestinal cells is known to reduce energy production by decreasing intracellular ATP concentration and arresting the cell cycle in mitosis. It also induces the production of reactive oxygen species (ROS) [39, 40]. This metabolic stress is likely to compromise mitochondrial function, promote apoptosis and destabilize tight junction proteins, providing a direct link between intracellular metabolic perturbations and barrier dysfunction [16, 41, 42].

Altered amino acid metabolism may have therapeutic relevance. Supplementation with protective amino acids (e.g., glutamine, threonine, serine, proline and cysteine) has been shown to enhance mucosal barrier integrity and reduce inflammation [43, 44]. Such strategies could mitigate MPA‐induced epithelial damage. This hypothesis warrants further investigation and could help determine the causal relationship between epithelial permeability and metabolic injury.

### 4.3 Limitations and Perspectives

While Caco‐2 cells are a widely accepted enterocyte model, they do not fully capture the complexity of the human intestinal epithelium, including goblet cells, immune components and microbial interactions. Moreover, MPAG hydrolysis and MPA‐associated toxicity predominantly occur in the colon, which represents an additional limitation of the present study based on a small‐intestinal epithelial cell model. Future studies using co‐cultures (e.g., Caco‐2/HT‐29) or organoids, coupled with modulation of microbial β‐glucuronidase activity, will provide greater translational relevance and may be used as a platform to identify and/or test metabolic interventions to preserve gut barrier integrity in patients receiving MPA.

## 5 Conclusion

This study provides novel mechanistic insights into MPA‐induced enteropathy by linking barrier dysfunction with metabolic stress in gut epithelial cells. Early MPA exposure of cells in vitro affects TEER and permeability, leading to persistent barrier dysfunction. Simultaneously, it induces metabolic rewiring, including alterations in nucleotide synthesis, carbohydrate consumption and amino acid metabolism, which collectively exacerbate epithelium vulnerability.

These findings suggest therapeutic interventions, such as metabolic supplementation or strategies to support epithelial cell renewal, to prevent or mitigate gastrointestinal toxicity in transplant patients.

## Author Contributions

C.B., P.V., P.M. and R.L. designed the study, contributed to planning and methodology, performed data analysis and drafted the manuscript. C.B., F‐L.S., C.V‐S. and V.C. conducted the experiments. F‐L.S. developed mass spectrometry techniques. C.V‐S., V.C. and P.V. developed methods to assess gut epithelial barrier integrity assessment. All authors read and approved the final manuscript.

## Funding

The authors have nothing to report.

## Conflicts of Interest

The authors declare no conflicts of interest.

## Data Availability

The data that support the findings of this study are available from the corresponding author upon reasonable request.
